# Utility of the Short Physical Performance Battery for Determining Walking Independence in Patients With Stroke

**DOI:** 10.7759/cureus.83128

**Published:** 2025-04-28

**Authors:** Sota Kajiwara, Motoki Maruyama, Takuto Oikawa, Manabu Horikawa, Masahiro Sasaki

**Affiliations:** 1 Rehabilitation, Akita Cerebrospinal and Cardiovascular Center, Akita, JPN; 2 Rehabilitation Medicine, Akita Cerebrospinal and Cardiovascular Center, Akita, JPN

**Keywords:** cutoff value, rehabilitation, short physical performance battery, stroke, walking independence

## Abstract

Introduction

The Short Physical Performance Battery (SPPB) is a functional assessment tool comprising three components: balance, gait, and muscle strength. It is easier to administer than the Berg Balance Scale (BBS), which has been used to determine walking independence. However, the cutoff value of the SPPB for determining walking independence in patients with stroke remains unknown. Therefore, this study aimed to compare the utility and cutoff values of the SPPB and BBS for determining walking independence in patients with stroke.

Methods

A total of 301 patients with stroke (mean age: 71.8±12.3 years) who were admitted to the convalescent rehabilitation ward of our center between July 2021 and June 2024 were included in this study. The SPPB, BBS, Stroke Impairment Assessment Set (SIAS), Mini-Mental State Examination (MMSE), and Functional Ambulation Categories (FAC) were administered at discharge. Walking independence was defined as FAC ≥4, and non-independence was defined as FAC ≤3. Logistic regression analysis was performed to examine the relationship between walking independence and the SPPB and BBS, with adjustments for age, sex, body mass index, days since onset, history of stroke, SIAS, and MMSE as covariates. The cutoff values of the SPPB and BBS for determining walking independence were calculated by receiver operating characteristic curve analysis, and the areas under the curve (AUC) for both measures were compared. The significance level was set at 0.05.

Results

Of the 301 patients, 184 were classified as walking independent, and 117 were classified as non-independent. The SPPB (odds ratio (OR)=2.15; 95% confidence interval (CI): 1.69-2.73) and BBS (OR=1.14; 95% CI: 1.25-1.56) were significantly associated with walking independence at discharge (p<0.001). The cutoff values for walking independence were ≥10 points for the SPPB (AUC: 0.948, sensitivity: 0.940, and specificity: 0.855) and ≥49 points for the BBS (AUC: 0.945, sensitivity: 0.967, and specificity: 0.821), without significant difference in AUC.

Conclusions

The SPPB demonstrated high discriminative accuracy comparable to the BBS, indicating its usefulness as a simple and practical evaluation tool.

## Introduction

Given that previous studies have reported that approximately 75-90% of patients with stroke regain independent ambulation [1], achieving walking independence represents a key goal of stroke rehabilitation. Determining walking independence is primarily based on physical assessments of balance, gait, and muscle strength. The Berg Balance Scale (BBS) has been widely used to assess fall risk and determine walking independence [2-6]. The BBS focuses on balance function and is a reliable assessment tool [7,8]. However, it is time-consuming and cannot capture multiple aspects of physical function, such as walking speed and muscle strength.

The Short Physical Performance Battery (SPPB) is a comprehensive measure of physical function consisting of three components: balance, gait, and standing up. It is characterized by its simplicity and short time to perform [9,10]. The SPPB has been used as a screening tool for assessing physical function in older adults, such as predicting the risks of falls and diagnosing sarcopenia and frailty [11-16]. However, no reliable cutoff value of the SPPB for walking independence, particularly in patients with stroke, has been identified, and no international guidelines have been established in this regard. Patients with stroke exhibit highly individualized functional impairments, such as motor paralysis, sensory deficits, and balance impairments [17-19]. Therefore, more appropriate indicators are needed to accurately evaluate walking independence. The SPPB includes gait and muscle strength assessments in addition to balance assessments, thereby having the potential to provide a comprehensive perspective on gait independence. Additionally, the SPPB requires less time and fewer resources than the BBS, making it potentially more feasible for clinical application.

This study aimed to compare the usefulness of the SPPB and the BBS for determining walking independence in patients with stroke and investigate the cutoff value of the SPPB. The findings of this study can help determine whether the SPPB is an alternative or complementary assessment tool to the BBS. Additionally, the findings may contribute to determining walking independence and improving rehabilitation planning for patients with stroke.

## Materials and methods

Study design and subjects

This was a single-center, retrospective, cross-sectional study including patients with stroke aged ≥18 years who were admitted to the convalescent rehabilitation ward of the Akita Cerebrospinal and Cardiovascular Center, Akita, Japan, between July 2021 and June 2024. The exclusion criteria were subarachnoid hemorrhage, co-existence of other neuromuscular diseases, prior orthopedic diseases, discharge due to death, and missing data. This study was conducted following the Strengthening the Reporting of Observational Studies in Epidemiology (STROBE) guidelines.

Data collection

Basic data, such as age, sex, body mass index (BMI), days since onset, history of stroke, and type of stroke, were collected from electronic medical records. Motor paralysis was assessed using the lower extremity motor items of the Stroke Impairment Assessment Set (SIAS) [20], and cognitive function was assessed using the Mini-Mental State Examination (MMSE) [21]. All other items were assessed at discharge by physical therapists, occupational therapists, and nurses.

Physical performance assessment

The SPPB consists of three items: standing balance, gait, and chair rise. Each item is scored on a 5-point scale, with the total score ranging from 0 to 12 [9]. Higher scores indicate higher physical and balance functions. Standing balance is assessed by the time the patient can hold side-by-side, semi-tandem, and full-tandem standing positions. Gait is evaluated by the time required for a 4-m optimal walk. Chair rise is assessed by the time needed to stand up on a chair five times with maximum effort. The use of assistive devices is permitted only in the gait test. The BBS consists of 14 items, including static, quasi-dynamic, and dynamic balance tasks [2]. Each item is rated on a 5-point scale, ranging from 0 to 4, with a total score ranging from 0 to 56. The higher the score, the higher the physical and balance functions. The SPPB and BBS were evaluated at discharge by the physical therapists.

Gait assessment

Walking independence was assessed using the Functional Ambulation Categories (FAC) [22]. FAC ≥4 was defined as walking independence, whereas FAC ≤3 was defined as walking non-independence. The decision on walking independence was made by a single, responsible physician after discussions among the physician, nurses, and physical therapists.

Statistical analysis

Data normality was assessed using the Shapiro-Wilk test. Comparisons between the walking independent and non-independent groups were performed using Mann-Whitney's U test or the chi-squared test. Logistic regression analysis was performed to examine the relationship between walking independence (0: non-independent; 1: independent) and SPPB and BBS scores, with age, sex, BMI, days since onset, history of stroke, SIAS lower extremity motor items, and MMSE as covariates [23-26]. Receiver operating characteristic (ROC) curve analysis was performed to determine the cutoff values of the SPPB and BBS for walking independence, and the areas under the curve (AUC) of the SPPB and BBS were compared. Sensitivity analysis was performed using the same analysis in subjects capable of walking (FAC ≥3). Walking independence was defined as FAC ≥4, and logistic regression and ROC curve analyses were performed. The significance level was set at 0.05 for all analyses.

Ethical considerations

This study was conducted in accordance with the Declaration of Helsinki. Personal information was handled in a strictly confidential manner. An opt-out approach was used to obtain informed consent from the participants, and they were provided with written explanations. The participants were explicitly informed that they could withdraw from the study at any time without any consequences. The study was approved by the Ethics Review Committee of the Akita Cerebrospinal and Cardiovascular Center (approval number: 23-3).

## Results

**Figure 1:**
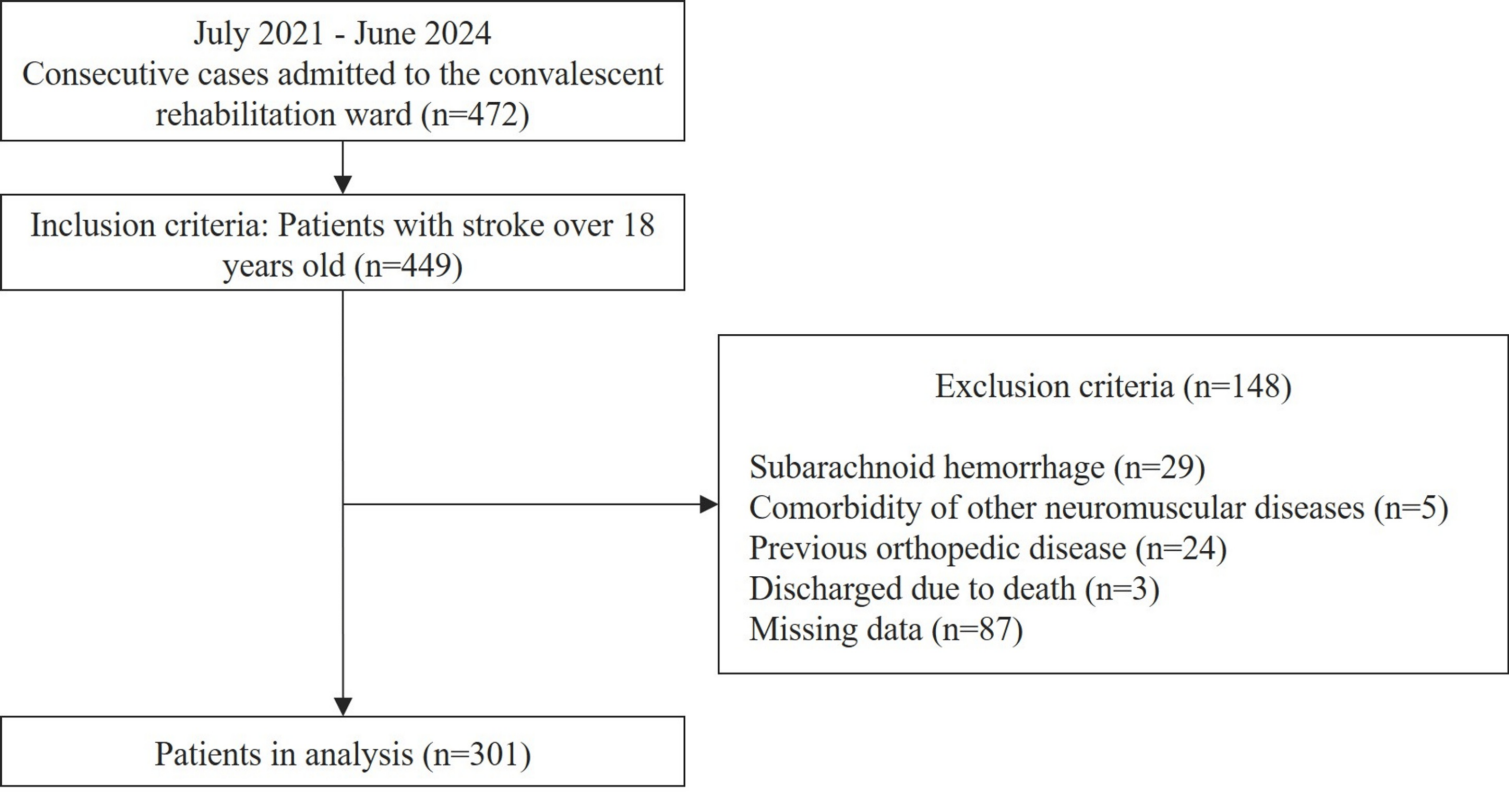
Flow diagram of the participant selection process.

A total of 449 patients were enrolled in this study. Among them, 301 patients with a mean age of 71.8±12.3 years met the inclusion criteria and were included in this study. Of the 301 patients, 184 (61.1%) were classified as independent walkers, and 117 (38.9%) were non-independent walkers. Figure 1 shows a flow diagram of the participant selection process.

Table 1 shows a comparison between the walking independent and non-independent groups. Significant differences in age (p<0.001), BMI (p<0.001), days since onset (p<0.001), history of stroke (p=0.003), SIAS lower extremity motor items (p<0.001), BBS (p<0.001), SPPB (p<0.001), and MMSE scores (p<0.001) were observed between the two groups.

**Table 1:** Comparisons between the walking independent and non-independent groups. Data are presented as median (interquartile range; Q1-Q3) or number (percentage). ^a^Mann-Whitney's U test ^b^Chi-squared test BMI: body mass index; SIAS: Stroke Impairment Assessment Set; BBS: Berg Balance Scale; SPPB: Short Physical Performance Battery; MMSE: Mini-Mental State Examination; FAC: Functional Ambulation Categories

Variables	Total, n=301	Independent walking group, n=184 (61.1%)	Non-independent walking group, n=117 (38.9%)	Test statistic	P-value
Age, years	74 (64-81)	71 (58.5-79)	78 (71-83)	U=14569.5	<0.001^a^
Sex (male)	178 (59.1)	109 (59.2)	69 (59)	X^2^=0	1.000^b^
BMI, kg/m^2^	21.9 (20.2-24.2)	22.4 (20.6-24.9)	21.1 (19.4-23.6)	U=8244	<0.001^a^
Stroke subtype					
Ischemia	194 (64.5)	120 (65.2)	74 (63.2)	X^2^=0.05	0.822^b^
Hemorrhage	107 (35.5)	64 (34.8)	43 (36.8)		
History of stroke	55 (18.3)	24 (13)	31 (26.5)	X^2^=7.789	0.005^b^
Days since onset, days	87 (57-116)	79 (51.5-110)	103 (72-124)	U=13455	<0.001^a^
SIAS lower extremity	12 (9-15)	15 (12-15)	9 (5-12)	U=4732	<0.001^a^
BBS, points	53 (42-56)	56 (54-56)	37 (11-45)	U=1176	<0.001^a^
SPPB, points	11 (5-12)	12 (11.5-12)	4 (0-8)	U=1124.5	<0.001^a^
MMSE, points	27 (22-30)	29 (26-30)	22 (18-26)	U=4244	<0.001^a^
FAC, score	4 (3-5)	5 (4-5)	2 (1-3)	U=0	<0.001^a^

**Figure 2:**
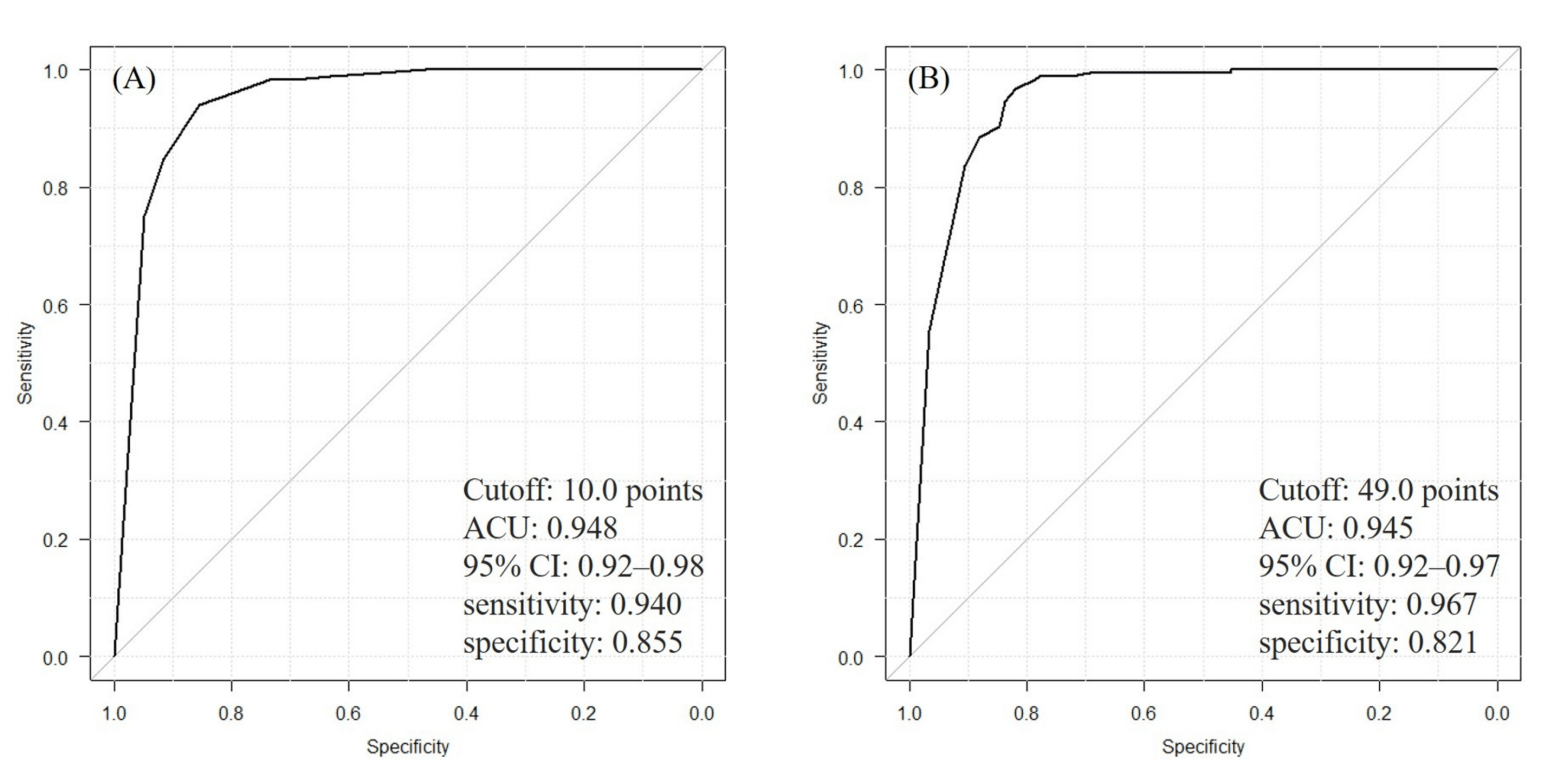
Receiver operating characteristic curves for the SPPB and BBS. (A) The cutoff value of the SPPB for determining walking independence was 10 points (sensitivity: 0.940; specificity: 0.855; AUC: 0.948). (B) The cutoff value of the BBS for determining walking independence was 49 points (sensitivity: 0.967; specificity: 0.821; AUC: 0.945). No significant difference in AUC was observed between the SPPB and BBS (p=0.842). BBS: Berg Balance Scale; SPPB: Short Physical Performance Battery; AUC: area under the curve; CI: confidence interval

Tables 2-3 and Figure 2 show the results of logistic regression and ROC curve analyses in the primary analysis. The logistic regression analysis revealed that the SPPB (OR=2.15; 95% CI: 1.69-2.73) and BBS (OR=1.14; 95% CI: 1.25-1.56) were significantly associated with walking independence at discharge (p<0.001), even after adjusting for covariate effects. The ROC curve analysis revealed that the cutoff values for walking independence were ≥10 points for the SPPB (AUC: 0.948, sensitivity: 0.940, and specificity: 0.855) and ≥49 points for the BBS (AUC: 0.945, sensitivity: 0.967, and specificity: 0.821). No significant difference in AUC was observed between the SPPB and BBS (p=0.84).

**Table 2:** Logistic regression analysis examining the relationship between walking independence and SPPB scores. Dependent variable is walking independence (0: non-independent; 1: independent). OR: odds ratio; CI: confidence interval; SE: standardized error; VIF: variance inflation factor; SPPB: Short Physical Performance Battery; BMI: body mass index; SIAS: Stroke Impairment Assessment Set; MMSE: Mini-Mental State Examination

Variables	OR	95% CI	SE	P-value	VIF
(Intercept)	0.03	0.00-35.7	3.59	0.334	-
SPPB	2.15	1.69-2.73	0.12	<0.001	1.32
Age	0.94	0.89-0.99	0.03	0.016	1.48
Sex	-	-	-	-	-
Male (reference)	-	-	-	-	-
Female	1.99	0.80-4.94	0.46	0.137	1.05
BMI	1.04	0.89-1.23	0.08	0.603	1.11
Days since onset	0.99	0.98-1.00	0.01	0.145	1.70
History of stroke	0.81	0.27-2.39	0.55	0.696	1.12
SIAS lower extremity	1.00	0.83-1.19	0.09	0.961	1.70
MMSE	1.06	0.99-1.14	0.04	0.106	1.16

**Table 3:** Logistic regression analysis examining the relationship between walking independence and BBS scores. Dependent variable is walking independence (0: non-independent; 1: independent). OR: odds ratio; CI: confidence interval; SE: standardized error; VIF: variance inflation factor; BBS: Berg Balance Scale; BMI: body mass index; SIAS: Stroke Impairment Assessment Set; MMSE: Mini-Mental State Examination

Variables	OR	95% CI	SE	P-value	VIF
(Intercept)	0.00	0.00-0.00	4.78	<0.001	-
BBS	1.40	1.25-1.56	0.06	<0.001	1.26
Age	0.98	0.93-1.03	0.03	0.448	1.57
Sex	-	-	-	-	-
Male (reference)	-	-	-	-	-
Female	2.24	0.90-5.59	0.47	0.085	1.05
BMI	1.09	0.93-1.28	0.08	0.267	1.11
Days since onset	1.00	0.98-1.01	0.01	0.434	1.69
History of stroke	0.42	0.15-1.19	0.53	0.102	1.10
SIAS lower extremity	1.02	0.86-1.22	0.09	0.787	1.55
MMSE	1.09	1.02-1.17	0.03	0.010	1.16

In the sensitivity analysis, 184 patients had FAC ≥4, whereas 55 had FAC=3. The logistic regression analysis results were consistent with those of the primary analysis (OR=1.99; 95% CI: 1.56-2.52; p<0.001). Although the ROC curve in the sensitivity analysis indicated a cutoff value of ≥11 points for walking independence, the discriminative accuracy remained high (AUC: 0.894, sensitivity: 0.848, and specificity: 0.818).

## Discussion

This study compared the utility of the SPPB and BBS for determining walking independence and defined the cutoff values in patients with stroke undergoing rehabilitation. The results showed an association between both the SPPB (OR=2.15; 95% CI: 1.69-2.73) and BBS (OR=1.14; 95% CI: 1.25-1.56) and walking independence at discharge, even after adjusting for the effects of motor paralysis and cognitive function. The cutoff values for the SPPB and BBS at discharge from the convalescent rehabilitation wards were ≥10 and ≥49 points, respectively. The SPPB demonstrated a discriminative ability similar to that of the BBS.

In this study, the degree of walking independence was comprehensively assessed by physicians, nurses, and physical therapists. The cutoff score of 49 points for the BBS reported in this study approximated the score of 45.5 points reported in a previous study [27], indicating the validity of the criteria for determining walking independence. The AUC of the SPPB and BBS were 0.948 and 0.945, respectively, and both demonstrated high accuracy in determining walking independence. These findings indicate that the SPPB has an evaluation capacity similar to that of the BBS. The balance between sensitivity (0.940) and specificity (0.855) at the cutoff value of the SPPB supports its applicability in clinical practice. Particularly, the high sensitivity and low false-negative rate highlight the ability of the test to accurately identify individuals capable of walking independently. Furthermore, the sensitivity analysis revealed that, even when limited to individuals capable of walking, the discriminative accuracy remained consistent, reinforcing the robustness of the results.

The SPPB has the advantage of reducing the burden on clinical sites that need to conduct evaluations efficiently with limited time and resources. Besides its simplicity, the SPPB has the ability to capture multiple aspects of physical function, such as balance, walking speed, and standing up, and is useful in predicting falls and diagnosing sarcopenia in older adults [11-16]. This study complements these previous studies and provides evidence that the SPPB is applicable to the assessment of walking independence in patients with stroke.

The identified cutoff value of ≥10 points in the SPPB carries important clinical significance. To achieve a total score of 10, patients must demonstrate relatively preserved physical performance. Specifically, scoring 10 points requires that at least one of the three subtests (balance, gait, or chair stand) be performed at the maximum level, which reflects well-compensated function in at least one domain. Moreover, the balance subtest demands that patients maintain a side-by-side stance for 10 seconds, a fundamental prerequisite for stable posture and safe gait initiation. In addition, the chair stand test requires patients to stand up from a chair five times without using their upper limbs for support, which indicates sufficient lower limb strength and dynamic balance. These conditions together suggest that a score of 10 represents not only a statistically meaningful cutoff but also a clinically relevant threshold for safe, independent ambulation in daily life. Therefore, this score may be useful for establishing rehabilitation goals and determining discharge readiness in convalescent stroke rehabilitation.

The BBS is a well-established tool that focuses on balance ability and has been widely used to assess fall risk and walking independence in patients with stroke [3,5,8]. Although the BBS has limitations in that it requires time and supplies for evaluation and does not consider other physical functions, such as walking speed and muscle strength, it can be used for a wide range of patients with severe to mild balance impairments. Therefore, the BBS can play an important role in tracking detailed changes in balance ability over time compared with the SPPB, which tends to show floor and ceiling effects [28,29].

The SPPB can be an alternative or complementary evaluation tool to the BBS. This method is practical and efficient for assessing walking independence in patients with stroke. Incorporating the SPPB cutoff value into clinical practice can help make rehabilitation goals and progress evaluations clearer and more efficient.

This study has some limitations. First, this was a single-center, retrospective study, and the assessment time point was limited to the time of discharge from the hospital. Second, this study was limited to patients admitted to the convalescent rehabilitation ward. Therefore, caution should be exercised when generalizing the results to outpatients or patients with mild conditions. Further studies are needed to determine the influence of using orthotics or walking aids or the type, lesion, and severity of stroke on the evaluation results.

## Conclusions

The SPPB and BBS cutoff values for walking independence were calculated at discharge in patients with stroke at the convalescent rehabilitation ward. The cutoff values were ≥10 and ≥49 points, respectively. The SPPB showed high accuracy in assessing walking independence, equivalent to that of the BBS, and was superior to the BBS in terms of simplicity and comprehensiveness. These findings highlight the potential of the SPPB to be widely adopted and clinically applied. The appropriate use of the SPPB and BBS can enable rehabilitation tailored to the various needs of patients with stroke and improve their quality and efficiency.
